# Reconstructing the Rasch-Built Myotonic Dystrophy Type 1 Activity and Participation Scale

**DOI:** 10.1371/journal.pone.0139944

**Published:** 2015-10-20

**Authors:** Mieke C. E. Hermans, Janneke G. J. Hoeijmakers, Catharina G. Faber, Ingemar S. J. Merkies

**Affiliations:** 1 Department of Neurology, Maastricht University Medical Center, Maastricht, the Netherlands; 2 Department of Neurology, Spaarne Hospital, Hoofddorp, The Netherlands; University of Valencia, SPAIN

## Abstract

**Introduction:**

A previously published Rasch-built activity and participation scale specifically designed for patients with myotonic dystrophy type 1 (DM1) was criticized for having been constructed in a relatively small cohort of patients and containing items too broadly phrased for DM1 patients, thus hampering its clinical use.

**Methods:**

We report the results of the reconstructed Rasch-built DM1 activity and participation scale for clinical use (DM1-Activ^C^) through Rasch analyses using an expanded questionnaire containing 146 more simply phrased activity and participation inquiries completed by 340 patients with DM1.

**Results:**

Through stepwise investigation including data quality control, model fit, response category ordering, local dependency and item bias, we succeeded in reconstructing the DM1-Activ^C^ consisting of 25 items that showed good Rasch model fit, including construct convergent validity, items’ weights and persons’ locations reliability, and unidimensionality.

**Conclusion:**

The DM1-Activ^C^ scale has been reconstructed and fulfills all modern clinimetric requirements. Its use is recommended in future longitudinal trials in patients with DM1 to determine its responsiveness.

## Introduction

At current stage, treatment of patients with myotonic dystrophy type 1 (DM1) is almost exclusively limited to symptomatic therapies with no therapeutic interventions to reverse of slow down the progression of the illness. However, since genetically based therapeutic treatments are emerging, solid outcome measures are needed to capture possible relevant clinical changes in patients with DM1 being exposed to these new therapies [1, 2].

Previously, we have described the first Rasch-built activity and participation measure specifically designed for patients with DM1 (DM1-Activ) [3, 4]. The DM1-Activ fulfilled all model requirements and is thus far the only interval level metric that has been extensively evaluated in this condition. However, this scale was criticized since its construction was only based on 168 patients, which might influence model robustness. Also, some items were vaguely defined and the response options were initially too broad, which could lead to misinterpretation (inability to discriminate between the response options) in patients with DM1. Although the paper was well-received, we were encouraged by the reviewers to reconstruct the scale in a much larger pool of patients to obtain a more robust model for use in future DM1 clinical studies [3].

Therefore, we have reconstructed the DM1-Activ for clinical use (DM1-Activ^C^), using a larger sample of patients with DM1 and a much broader pool of items representing the Activities and Participation component of the International Classification of Functioning, Disability and Health (ICF 2001) [5].

## Methods

### Participants

Patients older than 18 years with genetically proven DM1 were recruited through the Dutch neuromuscular patients’ association. The protocol was approved by the Medical Ethics Committee of the Maastricht University Medical Center. Written informed consent was obtained from all participants. A total of 340 patients with DM1 were included, fulfilling optimal sample size requirements for scale construction [6].

### Questionnaire development

The questionnaire was constructed as previously described [3]. In addition to the 49 previously selected items from the pre-phase DM1-Activ scale, we collected extra activity and participation items from the WHO-ICF items list [1, 5]. The preliminary DM1-Activ^C^ scale contained 146 items that provide information about activities and participation, phrased in a simplistic, short and unambiguous way. Response options were: unable to perform (0), able to perform, but with difficulty (1), and able to perform, without any difficulty (2) and were based on the previously determined discriminative ability of DM1 patients [3]. An item was scored (3) if it was ”not applicable” to the patient. Stepwise instructions were given to patients for completion of the questions (see S1 Fig for an overview of the guidelines/instructions).

The preliminary DM1-Activ^C^ was completed by 340 patients. A random sample of 223 patients completed the questionnaire ~4 weeks later for test-retest reliability studies. The original 20-item DM1-Activ scale was also completed separately to provide convergent evidence for construct validity.

### Statistics

#### Rasch analyses

Obtained preliminary DM1-Activ^C^ data were subjected to Rasch analyses to determine whether model expectations were met. Several aspects were addressed including fit statistics, ordered thresholds, local independency, differential item functioning (item bias: DIF) and unidimensionality. Item bias was checked on personal factors: age (<30 years, 30–50 years, >50 years), gender, diagnosis phenotype (mild type, adult type, childhood/congenital type), and degree of education (elementary school, high school, university) as was previously applied [3]. Items or patients not fulfilling these requirements were removed or adjusted to obtain good model fit, hereby creating an interval scale [3, 4, 7, 8].

#### Reliability and Validity studies

Internal consistency reliability of the final DM1-Activ^C^ scale was determined by calculating the Person Separation Index (PSI). Moreover, test-retest reliability studies were performed to investigate consistency of item difficulty hierarchy and patient ability locations. Reliability was quantified by calculation of the intraclass correlation coefficient using ANOVA for group comparison and expressed as R^2^: the fraction of variance [9, 10]. The external construct convergent validity of the DM1-Activ^C^ scale was tested by correlations with the original 20-item DM1-Activ scale, using the obtained Logits scores from both scales [3].

Further analyses were undertaken using Stata Statistical Software version 12.0 with Bonferroni corrections if needed and SigmaPlot Software [11].

## Results

### General aspects and data quality control

The basic characteristics of the 340 patients are presented in Table 1. As part of data quality control, items with >10% missing values (n = 29) and items with inadequate face validity (n = 12) according to DM1 expert’s opinion (CF, MH) were removed. In the model construction, items scored as (3) “not applicable” were interpreted as missing data. Also, 28 patients were removed prior to Rasch analyses (16 without age record, 6 with unknown phenotype classification, 3 with unknown gender, 2 with >10% uncompleted items, and 1 with unknown educational level). A total of 105 items and 312 patient records were finally subjected to Rasch analyses, continuously checking the distribution of persons within the class intervals.

**Table 1:** Basic characteristics of patients with DM1.

Number of patients		340
Gender (%)	Female	49.7%
	Male	50.3%
Age (years)	mean (SD), range	47.5 (12.5), 18–82
Diagnosis type (%)	Mild	12.1%
	Adult	77.3%
	Child/congenital	10.6%
Educational level (%)	Elementary school	15.9%
	High school	69.4%
	University	14.7%

### Rasch analysis of the pre-phase DM1-Activ^C^ scale

The preliminary 105-items DM1-Activ^C^ showed overall misfit. The mean residual for items was -0.085 (SD 1.213) and for persons -0.278 (SD 1.361), indicating reasonable model fit. However, the item-trait chi-square probability was significant (P<0.00001; degrees of freedom (DF) 420), indicating lack of invariance. No disordered thresholds were seen. A proportion of 0.14 (95% CI 0.11–0.17) of the t-tests performed fell outside the ±1.96 range, indicating multidimensionality.

### Fitting data to the Rasch model

The individual item fit statistics of 32 items demonstrated model misfit (having significant chi-square probability and/or fit residuals exceeding ±2.5) and were removed one by one (73 items remaining).

Local dependency was seen between many items’ residuals (defined as Spearman’s correlation coefficient ≥0.3). Starting with the highest residual correlations (≥0.7, then ≥0.6, through to ≥0.3), the item of each residual correlating set showing the least face validity according to experts’ opinion (CF, MH) or having over- or underdiscrimination on its category probability curve, was removed (45 items removed stepwise; 28 items remaining).

One item showed item bias (uniform DIF) on age and diagnosis phenotype and two items had non-uniform DIF on diagnosis phenotype. All 3 items were removed. Hence, at this stage 25 items were kept. However, the item “able to run” demonstrated uniform DIF on age. This item was one of the most difficult activities to perform. Since we aimed to obtain a wide range of measurement with a proper targeting of patient locations by the items’ thresholds, we decided to keep this item in the model. However, before splitting this item and creating 2 subsets (able to run for age <30 years versus able to run for ages ≥30 years), a test for unidimensionality was performed, since RUMM2030 software does not provide the opportunity to do this after splitting an item.

### Clinimetric properties of the final DM-Activ^C^

The final 25-item DM1-Activ^C^ met all Rasch model’s expectations (Table 2). The mean fit residual for items was -0.270 (SD 0.920) and for persons -0.273 (SD 0.773). The overall item-trait interaction chi-square probability was non-significant (P = 0.39; DF 104), indicating the required property of invariance. The independent t-tests between person estimates from 7 most “positive loaded” versus 7 most “negative loaded” items demonstrated a proportion of 0.067 (95% CI 0.043–0.091) falling outside the ±1.96 range, suggesting acceptable unidimensionality. Subsequently, the item “able to run” was split into 2 separate age-dependent items.

**Table 2:** DM1- Activ^C^ calibration.

Item	Location	SE	Residual^a^	P-value^b^
Are you able to…				
eat soup?	-3.305	0.198	-0.185	0.610198
visit family or friends?	-2.967	0.176	-0.113	0.653494
care for your hair and body?	-2.608	0.18	-0.136	0.793144
dress your lower body?	-2.314	0.162	-0.314	0.532921
wash your upper body?	-2.278	0.164	0.076	0.933869
take a shower?	-2.222	0.162	-0.91	0.422836
wash your lower body?	-2.15	0.16	-0.549	0.80354
get out of bed?	-1.777	0.151	1.836	0.003088
move a chair?	-1.217	0.143	-0.609	0.811204
do the dusting/cleaning?	-1.071	0.145	-0.743	0.514443
do the shopping?	-0.825	0.137	0.818	0.452093
tie the laces of your shoes?	-0.569	0.134	1.727	0.218672
catch an object (e.g. a ball)?	-0.527	0.134	0.268	0.282685
use dustpan and brush?	-0.145	0.132	-0.821	0.605114
empty dustbin?	-0.013	0.127	-1.434	0.294051
make up your bed?	0.491	0.128	-0.238	0.740776
vacuum clean?	0.807	0.124	-1.127	0.195076
serve coffee/tea on a tray?	1.408	0.121	-0.918	0.587366
dance?	1.844	0.125	0.149	0.08681
stand up from squatting position?	2.162	0.129	0.534	0.580312
stand on one leg?	2.287	0.126	1.141	0.100675
run (***<30 years***)?	2.343	0.486	-2	0.823465
walk uphill?	2.399	0.133	-1.085	0.628618
walk 3 flights of stairs?	2.832	0.134	-1.114	0.140408
carry and put down heavy object (10 kg)?	3.509	0.139	-0.674	0.244503
run (***≥ 30 years***)?	3.904	0.148	-0.599	0.709763

SE: standard error

^a^Residuals summarize the deviation of observed from expected responses and lie within the recommended range of -2.5 and +2.5.

^b^Bonferroni corrected probability scores were not significant (Bonferroni cut-off: p<0.000385)), indicating no deviation from the model expectations.

In the final DM1-Activ^C^ scale, item “able to eat soup” (-3.305 logits) was the easiest item while “able to run” for patients ≥30 years (3.904 logits) was the most difficult item (Table 2). Two patients (0.6%) demonstrated most severe activity limitations or participation restrictions (floor effect), while 23 patients (7.4%) with no symptoms had a maximum score (ceiling effect). A guideline for the completion of the DM1-Activ^C^ is presented in S1 Fig.

### External construct validity and reliability studies

**Fig 1:**
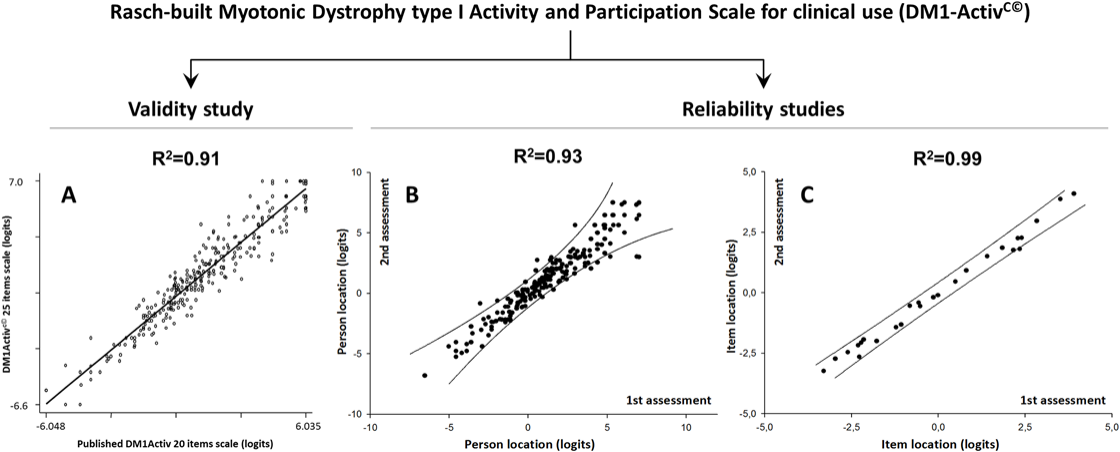
External construct convergent validity and test-retest reliability for DM1-Activ^C^. The 25-item DM1-Activ^C^ scale demonstrates high external construct/convergent validity with the 20-items DM1-Activ (A). Patients’ ability (B) and items’ difficulty hierarchy (C) in the first and second assessment are located within the 95% confidence intervals indicating ideal invariance (solid lines).

The final 25-item DM1-Activ^C^ scale demonstrated high external construct convergent validity (R^2^ = 0.91 correlation with the original 20-items DM1-Activ; p<0.0001; Fig 1A). Internal reliability was robust (PSI = 0.95). The test-retest reliability for patient’s location and item’s difficulty was good (R^2^ = 0.93 and R^2^ = 0.99, respectively; p<0.0001; Fig 1B and 1C). All items and almost all patient locations were within the 95% CI lines, reflecting ideal invariance.

## Discussion

In the current study, the reconstructed DM1-Activ^C^ outcome measure for activity limitations is presented (S1 Fig). This 25-items interval scale has been specifically designed for patients with DM1 and fulfilled all Rasch model expectations, with robust validity and reliability aspects. The internal reliability (PSI) of the DM1-Activ^C^ scale was high, indicating discriminative ability of the scale to differentiate between patients with various levels of disability [9]. Its responsiveness is currently being examined as part of an international multi-center randomized controlled trial: OPTMISTIC, the Observational Prolonged Trial in myotonic dystrophy type 1 to Improve Quality of Life Standards, a Target Identification Collaboration. However, consensus should also be met within the DM1 community regarding whether responsiveness should be based on statistical differences or implementing the concept of minimum clinically important difference (MCID) using published practical guidelines [12] or applying the MCID concept using the varying standard errors across the scale’s continuum, as has been demonstrated in various chronic neurological conditions [13, 14].

Compared to the previously published 20-item DM1-Activ scale [3], the current reconstructed DM1-Activ^C^ contains 5 more items with a more stable model pattern. The item difficulties of the DM1-Activ^C^ cover a wider range than the original DM1-Activ (7.209 logits versus 6.15 logits), thus providing a better targeting of patients. This was also reflected in a lower percentage of ceiling effect in DM1 patients (7.4% versus 8.6%) [3]. In addition, the final 25 items of the DM1-Activ^C^ represent the whole spectrum of abilities of the preliminary 146 items. Item “able to eat soup” was initially among the 9 easiest items, whereas “able to run” was initially one of the most difficult items to perform of all 146 items. Therefore, we believe that the final 25 items of the DM1-Activ^C^ represent a wide range of daily and social activities. Finally, for the construction of DM1-Activ^C^ a larger number of patients was examined having elementary school as their highest educational degree (in the current study: n = 54 patients; in the original DM1-Activ this was only n = 19 patients). This implies that the final DM1-Activ^C^ model findings represents more appropriately patients with DM1 having all levels of study degree (elementary, high school, or university), since the final 25-items did not show any item bias on personal factor educational level.

There are some methodological issues that should be addressed. First, despite the strong correlation between the DM1-Activ^C^ and the original DM1-Activ, further studies are currently performed investigating the impact of DM1 from a more holistic point of view: from person factors that might influence scoring (CTG repeat length, socio-economic status (e.g., marital status, working and financial income), amount of family support or number of family members affected, duration of illness) to the impact of impairments such as e.g. fatigue, sleep disturbances, pain, apathy, cognitive dysfunction, depression, cardiac involvement, and having a pacemaker or a defibrillator implanted or not, that might influence daily/social functioning as assessed with DM1-Activ^C^. These efforts are currently being performed at our center through an initiated national registry and follow up study, and as part of the ongoing international collaborative OPTIMISTIC longitudinal trial including examining the responsiveness of DM1-Activ^C^ [15–17]. Second, data on the exact repeat sizes, especially if repeat sizes are larger than 150 repeats, are not generally available, as this is not part of the routine DNA tests in the Netherlands. Moreover, though CTG repeat length is correlated with age at onset, there is considerable heterogeneity of symptoms shown by patients with similar CTG repeat sizes, partly explained by the presence of somatic mosaicism and somatic expansion over time. However, we sincerely belief that the group examined represents a clinically well-defined cohort of patients with DM1, but the influence of CTG repeat length on DM1Avtiv^C^ scoring needs to be determined in future studies. Third, a total of 52% of the original DM1-Activ findings could be explained by muscle weakness, suggesting that other factors contribute to activity limitations and participation restrictions. Of particular interest would also be to study the discriminatory ability of the DM1-Activ^C^ using the generally applied muscular impairment rating scale staging [18]. We believe that these findings will help the DM1 community understand the translational impact of various impairments explaining activity limitations and participation restrictions as captured by DM1-Activ^C^. However, these studies were not part of the scope of the current paper. In the current study, the most practical available factors have been examined. Fourth, while daily and social activities are not primarily dependent on someone’s health status (i.e., being healthy or having a particular underlying illness), the difficulty to execute a specific daily task (i.e. the corresponding weight of an item) is certainly disease specific. Therefore, it is not surprising that the difficulty estimates of DM1-Activ^C^ items differ from similar items in other neuromuscular Rasch-built measures like the ACTIVLIM (Rasch-built measure of activity limitations in children and adults with neuromuscular disorders), I-RODS (Rasch-built Overall Disability Scale for immune-mediated peripheral neuropathies) or R-Pact (Rasch-built Pompe-specific activity scale) [19–21]. Similar observations have been reported examining manual ability in several diagnostic groups, stating that most of the manual item difficulties were disease-dependent emphasizing the danger of using generic scales without prior investigation of item invariance across diagnostic groups [22]. Efforts should be made to inform the DM1 community about the value of disease-specific outcome measures, which should preferably be interval rather than ordinal scales, since the categories of the latter have no numerical value [23–25]. Fifth, since DM1 is considered a rare disorder, it is expected that most studies will be internationally multi-center based. Therefore, future studies should focus on determining the cross-cultural validity of the constructed DM1-Activ^C^ [26]. Through the ongoing OPTIMISTIC trial, additional data will be gathered to examine the cross-cultural aspects of the DM1-Activ^C^. Finally, other factors like duration of complaints might be of influence in completing the DM1-Activ^C^ items. In the current study, “duration” as a person factor could not be used, since the data were incomplete on this factor. However, in the original paper, no item bias on duration was seen [3].

In conclusion, the DM1-Activ^C^ scale fulfills all modern clinimetric requirements, and its use is recommended in future longitudinal trials in patients with DM1 to determine its responsiveness.

## Supporting Information

S1 Fig Instruction to complete the DM1-Activ^C^. For further instructions to translate the obtained ordinal scores to an interval (Logits) scores, please contact the authors.
(PDF)

S1 Appendix Raw data. Legend to data: Studynr = study number; gender (0 = female; 1 = male); agecat = age category (1 = age<30 years; 2 = age≥30 and age<50 years; 3 = age≥50 years); diagnosis = diagnosis type (1 = mild; 2 = adult; 3 = child/congenital); studydegr = study degree (1 = elementary school; 2 = high school; 3 = university). Description items preliminary DM1-Activ^C^ (0 = impossible to perform; 1 = able to perform, but with difficulty; 3 = able to perform, without difficulty): r_ods001 = bend and pickup; r_ods002 = stand <15 minutes; r_ods003 = stand for hours; r_ods004 = stand on one leg; r_ods005 = stand up from sitting; r_ods006 = stand up from lying; r_ods007 = get out of bed; r_ods008 = stand up from squatting position; r_ods009 = kneel down; r_ods010 = sit down from standing; r_ods011 = lie down from standing; r_ods012 = walk indoor; r_ods013 = walk up 1 stair; r_ods014 = walk up 2 stairs; r_ods015 = walk up 3 stairs; r_ods016 = walk upstairs with a bag; r_ods017 = jump; r_ods018 = run; r_ods019 = walk outdoor <100 meters; r_ods020 = walk outdoor <1 kilometers; r_ods021 = walk outdoor >1 kilometers; r_ods022 = walk uphill; r_ods023 = walk in the woods; r_ods024 = walk in the dunes; r_ods025 = walk downstairs; r_ods026 = walk on even ground; r_ods027 = walk on uneven ground; r_ods028 = walk avoiding obstacles; r_ods029 = dance; r_ods030 = travel by public transport; r_ods031 = travel by train; r_ods038 = open/close the door; r_ods039 = turn a key in a lock; r_ods040 = open a door with a key; r_ods041 = open an upper window; r_ods042 = open a low window; r_ods043 = carry a tray; r_ods044 = serve coffee/tea on a tray; r_ods047 = put down a mug/glass; r_ods051 = hang up a coat; r_ods052 = lift heavy object (10 kilograms); r_ods053 = carry/put down a heavy object; r_ods054 = move a table; r_ods055 = move a chair; r_ods056 = pick up a small object; r_ods057 = handle small objects (e.g. a coin); r_ods058 = catch an object (e.g. a ball); r_ods059 = throw an object (e.g. a ball); r_ods060 = wash your upper body; r_ods061 = wash your lower body; r_ods062 = wash your face; r_ods063 = wash your entire body; r_ods064 = take a shower; r_ods066 = dry your body; r_ods067 = brush your teeth; r_ods068 = care your hair/body; r_ods069 = go to the toilet; r_ods071 = clip your finger nails; r_ods072 = clip your toe nails; r_ods076 = dress your upper body; r_ods077 = dress your lower body; r_ods078 = put on a sweater; r_ods079 = put on a T-shirt; r_ods080 = button shirt/blouse; r_ods081 = zip trousers; r_ods082 = put on a coat; r_ods083 = put on gloves; r_ods084 = put on shoes; r_ods085 = tie laces; r_ods086 = eat; r_ods087 = drink out of a mug/glass; r_ods088 = use a knife/fork(spoon); r_ods089 = eat soup; r_ods090 = peel a banana; r_ods091 = peel an apple/orange; r_ods092 = mop the floor; r_ods094 = vacuum; r_ods099 = clean the toilet; r_ods102 = put away the dishes; r_ods104 = put the laundry in the washing machine; r_ods108 = empty the dustbin; r_ods109 = put rubbish outside; r_ods110 = use a dustpan and brush; r_ods111 = do dusting; r_ods113 = make up the bed; r_ods114 = change sheets; r_ods115 = do the cooking; r_ods116 = make a sandwich; r_ods117 = slice vegetables; r_ods118 = make coffee/tea; r_ods119 = go to the supermarket; r_ods120 = do shopping; r_ods121 = carry shopping; r_ods124 = go to the general practitioner; r_ods125 = go to the hospital; r_ods128 = get money from the cashpoint; r_ods129 = fill in a form; r_ods136 = go to a party; r_ods137 = go out for dinner; r_ods139 = visit family/friend; r_ods140 = visit neighbors; r_ods141 = work on a computer; r_ods142 = read a book; r_ods143 = read a newspaper; r_ods144 = make a telephone call. Empty cells are missing data.
(XLS)

## References

[1] Streiner DL, Norman GR. Health measurement scales. A practical guide to their development and use New York: Oxford University Press; 1995.

[2] Magana JJ, Cisneros B. Perspectives on gene therapy in myotonic dystrophy type 1. J Neurosci Res. 2011;89(3):275–85. 10.1002/jnr.22551 21259315

[3] Hermans MC, Faber CG, De Baets MH, de Die-Smulders CE, Merkies IS. Rasch-built myotonic dystrophy type 1 activity and participation scale (DM1-Activ). Neuromuscul Disord. 2010;20(5):310–8. 10.1016/j.nmd.2010.03.010 20363134

[4] Rasch G. Probabilistic models for some intelligence and attainment tests Chicago: University of Chicago Press; 1980.

[5] World Health Organization. International classification of functioning, disability and health.2001; Geneva.

[6] Linacre JM. Sample size and item calibration stability. Rasch Measure Trans. 1994;7:28.

[7] Tennant A, Conaghan PG. The Rasch measurement model in rheumatology: What is it and why use it? When should it be applied, and what should one look for in a Rasch paper?. Arthritis Rheum. 2007;57:1358–62. 18050173 10.1002/art.23108

[8] Pallant JF, Tennant A. An introduction to the Rasch measurement model: an example using the Hospital Anxiety and Depression Scale (HADS). Br J Clin Psychol. 2007;46(Pt 1):1–18. 17472198 10.1348/014466506x96931

[9] Fisher WP. Reliability statistics. Rasch Measure Trans. 1992;6:238.

[10] Wright BD, Stone MH. Best test design Chicago: Mesa Press; 1979.

[11] Bland JM, Altman DG. Multiple significance tests: the Bonferroni method. BMJ. 1995;310(6973):170 7833759 10.1136/bmj.310.6973.170 PMC2548561

[12] Sloan JA. Assessing the minimally clinically significant difference: scientific considerations, challenges and solutions. COPD. 2005;2(1):57–62. 17136963 10.1081/copd-200053374

[13] Hobart J, Cano S. Improving the evaluation of therapeutic interventions in multiple sclerosis: the role of new psychometric methods. Health Technol Assess. 2009;13(12):iii, ix-x, 1–177. 10.3310/hta13120 19216837

[14] Draak TH, Vanhoutte EK, van Nes SI, Gorson KC, Van der Pol WL, Notermans NC, et al Changing outcome in inflammatory neuropathies: Rasch-comparative responsiveness. Neurology. 2014;83(23):2124–32. 10.1212/WNL.0000000000001044 25378677

[15] Meola G, Cardani R. Myotonic dystrophies: An update on clinical aspects, genetic, pathology, and molecular pathomechanisms. Biochim Biophys Acta. 2015;1852(4):594–606. 10.1016/j.bbadis.2014.05.019 24882752

[16] Laberge L, Gagnon C, Dauvilliers Y. Daytime sleepiness and myotonic dystrophy. Curr Neurol Neurosci Rep. 2013;13(4):340 10.1007/s11910-013-0340-9 23430686

[17] Hermans MC, Merkies IS, Laberge L, Blom EW, Tennant A, Faber CG. Fatigue and daytime sleepiness scale in myotonic dystrophy type 1. Muscle Nerve. 2013;47(1):89–95. 10.1002/mus.23478 23042586

[18] Mathieu J, Boivin H, Meunier D, Gaudreault M, Begin P. Assessment of a disease-specific muscular impairment rating scale in myotonic dystrophy. Neurology. 2001;56(3):336–40. 11171898 10.1212/wnl.56.3.336

[19] Vandervelde L, Van den Bergh PY, Goemans N, Thonnard JL. ACTIVLIM: a Rasch-built measure of activity limitations in children and adults with neuromuscular disorders. Neuromuscul Disord. 2007;17(6):459–69. 17433675 10.1016/j.nmd.2007.02.013

[20] van Nes SI, Vanhoutte EK, van Doorn PA, Hermans M, Bakkers M, Kuitwaard K, et al Rasch-built Overall Disability Scale (R-ODS) for immune-mediated peripheral neuropathies. Neurology. 2011;76(4):337–45. 10.1212/WNL.0b013e318208824b 21263135

[21] van der Beek NA, Hagemans ML, van der Ploeg AT, van Doorn PA, Merkies IS. The Rasch-built Pompe-specific activity (R-PAct) scale. Neuromuscul Disord. 2013;23(3):256–64. 10.1016/j.nmd.2012.10.024 23273871

[22] Arnould C, Vandervelde L, Batcho CS, Penta M, Thonnard JL. Can manual ability be measured with a generic ABILHAND scale? A cross-sectional study conducted on six diagnostic groups. BMJ Open. 2012;2(6). 10.1136/bmjopen-2012-001807 23117570 PMC3533037

[23] Merbitz C, Morris J, Grip JC. Ordinal scales and foundations of misinference. Arch Phys Med Rehabil. 1989;70(4):308–12. 2535599

[24] Stevens SS. On the Theory of Scales of Measurement. Science. 1946;103(2684):677–80. 20984256

[25] Grimby G, Tennant A, Tesio L. The use of raw scores from ordinal scales: time to end malpractice? J Rehabil Med. 2012;44(2):97–8. 10.2340/16501977-0938 22334345

[26] Kucukdeveci AA, Sahin H, Ataman S, Griffiths B, Tennant A. Issues in cross-cultural validity: example from the adaptation, reliability, and validity testing of a Turkish version of the Stanford Health Assessment Questionnaire. Arthritis Rheum. 2004;51(1):14–9. 14872450 10.1002/art.20091

