# Supplementary material for: Reconstructing the Rasch-Built Myotonic Dystrophy Type 1 Activity and Participation Scale
Source: PLoS One. 2015 Oct 20;10(10):e0139944. doi: 10.1371/journal.pone.0139944 (PMC4618741; doi:10.1371/journal.pone.0139944)
Supplement: S1 Fig — For further instructions to translate the obtained ordinal scores to an interval (Logits) scores, please contact the authors. (PDF) [file pone.0139944.s001.pdf]

INSTRUCTIONS
to complete the DM1-ACTIV[C]©

DM1-ACTIV$^{C©}$
General aspects

- This 25 item questionnaire covers a wide range of daily and social activities and represents the preliminary 146 items based on the WHO-ICF classification of activities and participation from which it was derived.

- The DM1-ACTIV$^{C©}$ captures personal ability to execute a task where the patient is requested to determine whether he/she is able to complete such a task independently.

	Item	Impossible to perform (0)	Able to perform, but with difficulty (1)	Able to perform, without difficulty (2)
	Are you able to...			
1	eat soup?	☐	☐	☐
2	visit family or friends?	☐	☐	☐
3	care for your hair and body?	☐	☐	☐
4	dress your lower body?	☐	☐	☐
5	wash your upper body?	☐	☐	☐
6	take a shower?	☐	☐	☐
7	wash your lower body?	☐	☐	☐
8	get out of bed?	☐	☐	☐
9	move a chair?	☐	☐	☐
10	do the dusting/cleaning?	☐	☐	☐
11	do the shopping?	☐	☐	☐
12	tie the laces of your shoes?	☐	☐	☐
13	catch an object (e.g. a ball)?	☐	☐	☐
14	use dustpan and brush?	☐	☐	☐
15	empty dustbin?	☐	☐	☐
16	make up your bed?	☐	☐	☐
17	vacuum clean?	☐	☐	☐
18	serve coffee/tea on a tray?	☐	☐	☐
19	dance?	☐	☐	☐
20	stand up from squatting position?	☐	☐	☐
21	stand on one leg?	☐	☐	☐
22	walk uphill?	☐	☐	☐
23	walk 3 flights of stairs?	☐	☐	☐
24	carry and put down heavy object (10 kg)?	☐	☐	☐
25	run?	☐	☐	☐

DM1-ACTIV$^{C©}$
General aspects

- Patients should complete the questionnaire after having received written and verbal instructions.

- Make sure that the patient is able to complete the questionnaire in a comfortable and quiet room.

- The patient should be comfortably sitting.

- Encourage the patient to complete the questions by themselves. However, a partner, relative or friend may be of assistence if the patient is unsure or has communication problems.

- It takes approximately 3 minutes to complete the questionnaire.

DM1-ACTIV$^{C©}$
Instructions-I

- Make sure that the answers given are due to the impact of myotonic dystrophy type I, rather than another concomitant disease (e.g. arthritis).

- Ask the patient to rate the tasks as either "Impossible to perform", "Able to perform, but with difficulty" or "Able to perform, without difficulty".

- If it is the first time for the patient completing the DM1-ACTIV$^{C©}$, go through each question with the patient and ensure the patient that the interpretation of each question is left to his/her best personal judgement rather than a fixed way to interpret each question.

DM1-ACTIV^{C}©
Instructions-II

- Instruct the patient that there are no right or wrong answers. Encourage the patient to choose the response that best describes his/her situation.
- A partner, family or a carer may help in interpreting the tasks in the way that reflects the situation of the patient as accurate as possible.
- For example: “Able to do the shopping?” The patient may experience difficulties at various levels, which may lead to problems executing such a task. This may also differ from patient to patient.
- Problems executing this task may be due to physical impairment such as weakness leading to mobility problems, transportation limitations, financial handling problems, executive cognitive dysfunctions.

DM1-ACTIV$^{C©}$
Instructions-III

- In case of any doubt or addressing a task that is not relevant to the patient (e.g. “I never do vacuum cleaning”), the patient is requested to choose an answer to the best of his/her personal judgment of being able or not to perform such a task, with or without difficulty.

- If still unsure and if possible, you may observe the patient performing the task, or miming the task.

- When using a special device (for example adapted cutlery (spoon), special cooking utensils, cane, crutch, or walker) to complete a task, the patient is requested to score “Able to perform, but with difficulty”.

- However, if the patient is not able to execute a task despite using special devices, the patient should score “Impossible to perform”.

DM1-ACTIV$^{C©}$ Instructions-IV

- When some assistance is needed (for example support by a caregiver), to complete a task, the patient is requested to score “Able to perform, but with difficulty”.

- However, if the patient is not able to contribute in completing a task, the patient should score “Impossible to perform”.
